# Evaluation of selenoproteins and proinflammatory cytokines in L-thyroxine-induced hyperthyroid rats: effects of selenium supplementation

**DOI:** 10.20945/2359-4292-2024-0444

**Published:** 2025-04-18

**Authors:** Aysun Yoldas, Nurten Bahtiyar, Birsen Aydemir, Selmin Toplan

**Affiliations:** 1 Department of Biophysics, Istanbul Yeniyuzyil University, Faculty of Medicine, Istanbul, Turkey; 2 Department of Biophysics, Istanbul University-Cerrahpaşa, Cerrahpaşa Faculty of Medicine, Istanbul, Turkey; 3 Department of Biophysics, Sakarya University, Faculty of Medicine, Sakarya, Turkey

**Keywords:** Hyperthyroidism, selenium, cytokines, selenoproteins

## Abstract

**Objective:**

This study investigated the effects of selenium, which is known for its antioxidant and immune-supporting properties, on serum levels of thyroid function markers, selenoproteins, and proinflammatory cytokines in a model of hyperthyroidism.

**Materials and methods:**

A total of 48 Wistar albino rats were distributed into 6 groups: a control group; a hyperthyroid group (HT group); a group fed 0.5 mg/kg sodium selenite (Se 1 group); a group fed 1 mg/kg sodium selenite (Se 2 group); a hyperthyroid group fed 0.5 mg/kg sodium selenite (HT + Se 1 group); and a hyperthyroid group fed 1 mg/kg sodium selenite (HT + Se 2 group) added to standard fodder. Serum levels of interleukin (IL)-1β, IL-6, IL-18, tumour necrosis factor alpha (TNF-α), selenoprotein P (SelP), and glutathione peroxidase 1 (GPx1) were measured using ELISAs.

**Results:**

IL-Iβ, IL-6, IL-18, and TNF-α levels were increased, but selenium, GPx1, and SelP levels were decreased in the hyperthyroid group compared with those in the control group. Selenium and GPx1 levels were increased, but TNF-α levels were decreased in the HT + Se 1 group compared with those in the HT group. Selenium, SelP, and GPx1 levels were increased, but TNF-α, IL-6, and IL-18 levels were decreased in the HT + Se 2 group compared with those in the HT group.

**Conclusion:**

Our results suggest that appropriate doses of selenium may be effective at preventing inflammation and providing protection against oxidative stress in hyperthyroid rats.

## INTRODUCTION

Hyperthyroidism is characterized by reduced serum thyroid stimulating hormone (TSH) levels despite increased free thyroxine (FT4) and free triiodothyronine (FT3) levels. In addition to the element iodine (I), selenium is an important trace element that plays a role in thyroid metabolism (^1,2,3^). Selenium is required for the biological activity of the deiodinase enzyme, which converts the thyroxine (T4) hormone into the biologically active hormone triiodothyronine (T3) (^4^). Additionally, selenium is a cofactor of many enzymes in the human body and is an essential trace element that plays a role in many vital functions as an antioxidant. Selenium exerts its biological effects through these selenoproteins and is present at higher levels in the thyroid gland than in other tissues (^1,2^).

Thyroid hormones are necessary for normal B lymphocyte development and the humoral immune response (^5^). The physiological effects of thyroid hormones may be altered by glucocorticoids, somatostatin, and cytokines. Cytokines are important elements of the adaptive immune response to the activation and proliferation of macrophages and natural killer (NK) cells (^6,7^). Proinflammatory cytokines are produced by activated macrophages and interfere with inflammatory reactions. Among these cytokines, interleukin-1 (IL-1), interleukin-6 (IL-6), and tumour necrosis factor alpha (TNF-α) trigger inflammatory events (^7^). Interleukin-1 beta (IL-1β) is a member of the IL-1 family of cytokines. This cytokine is an important mediator of the inflammatory response and is involved in a variety of cellular activities, including cell proliferation, differentiation, and apoptosis. Interleukin-18 (IL-18), another member of the IL-1 family, is involved in the regulation of both innate and adaptive immunity. IL-18 promotes interferon-gamma (IFN-γ) production and activates NK and T cells. IL-6 is another cytokine that is responsible for stimulating acute phase protein synthesis and the production of neutrophils in the bone marrow. It supports the growth of B cells and is antagonistic to regulatory T cells (^7,8^). An in vitro experimental study has shown that cytokines can be produced by stimulating thyroid follicular cells with IL-1, IFN-γ and TNF-α (^9^).

Selenoprotein P (SelP) is a selenium-containing protein in plasma that acts as a carrier protein. It is responsible for the transfer and storage of selenium to tissues. SelP protects cells from peroxynitrite-mediated oxidation and nitration. It reduces phospholipid hydroperoxides in vitro and acts as an antioxidant (^10,11^). In addition, glutathione peroxidases (GPxs) constitute one of the most important selenoprotein families. GPxs catalyse the reduction reactions of hydrogen peroxidase and organic hydroperoxidases, ensuring the degradation of peroxides and their conversion to water. Glutathione peroxidase 1 (GPx1) is abundant in the cell cytoplasm, functions to prevent oxidative damage, and reduces its activity by reducing hydrogen peroxide (H_2_O_2_). H_2_O_2_ is an important signalling molecule involved in many mechanisms and cycles related to cell proliferation, apoptosis, the stress response and mitochondrial functions in organisms. Elevated H_2_O_2_ levels impair cellular signalling, and excessive GPx1 production may cause reductive stress. GPx1 is an important selenium reservoir. GPx1 levels decrease in individuals fed a low-selenium diet (^12^, ^13^).

In hyperthyroidism, the production of reactive oxygen species (ROS) increases, and changes in the antioxidant protective systems of various tissues occur (^14,15^). Trace amounts of selenium in biological systems act as radical oxygen scavengers, but at relatively high concentrations, selenium has a toxic effect on some systems (^4^).

In light of the aforementioned information, clinical and experimental studies in the literature have investigated the effects of selenium, either alone or in combination with other antioxidants, on thyroid hormones in individuals with hyperthyroidism. However, these studies have reported conflicting findings (^16,17,18^). Additionally, studies have examined selenium and selenoprotein levels, as well as the levels of proinflammatory cytokines, in individuals with hyperthyroidism (^19,20,21^). However, studies specifically investigating the effects of selenium supplementation on these biomarkers in individuals with thyroid disorders are scarce. Therefore, our study aimed to investigate the effects of supplementation with 2 different doses of selenium, which were used to determine the most appropriate selenium dose, on the levels of thyroid hormones, selenoproteins, and proinflammatory cytokines in rats with experimentally induced hyperthyroidism.

## MATERIALS AND METHODS

### Study design

In this experimental study, 48 male Wistar albino rats, each weighing 250 ± 15 g, were used. All the animals were housed under the same environmental conditions, i.e., at a room temperature of 25 °C with an artificial light cycle (lights: 08:00-20:00 h), and were allowed one week for adaptation. The animals were subsequently divided into 6 experimental groups: the control group; the hyperthyroid group (HT group); the group fed 0.5 mg/kg sodium selenite (Sigma-Aldrich, St. Louis, MO, USA) (Se 1 group); the group fed 1 mg/kg sodium selenite (Se 2 group); the hyperthyroid group fed 0.5 mg/kg sodium selenite (HT + Se 1 group); and the hyperthyroid group fed 1 mg/kg sodium selenite (HT + Se 2 group). The control group was fed standard rat fodder. Hyperthyroidism was induced by adding 4 mg/kg L-thyroxine (Sigma–Aldrich, T-2376) to standard fodder. All the animals drank tap water, and the experiment ended after 30 days (^15,22,23^). At the end of the experimental period, blood samples were collected via cardiac puncture. Blood samples were collected in tubes containing gel. Serum samples were removed from blood samples after centrifugation at 3,500 × g for 5 min and were stored in polyethylene Eppendorf tubes at −80 °C until analysis. Our protocol and methods were approved by the Animal Care and Use Committee of the Laboratory of Animal Service of Istanbul University, Istanbul, Turkey (Approval number: 37-2013).

### Measurement of thyroid hormone levels in serum samples

The serum levels of the thyroid hormones TSH, FT3, and FT4 were measured using ELISA kits (Sunred Biological Technology, Shanghai, China). All procedures were performed according to the manufacturer’s instructions.

### Measurement of selenium levels in serum samples

Serum selenium levels were measured using an inductively coupled plasma optical emission spectrophotometer (ICP-OES, Thermo iCAP 6000, Cambridge, UK). All the experimental materials were subjected to decontamination and demineralization by immersion in acid baths containing 10% nitric acid (HNO_3_) (Merck, Darmstadt, Germany) for 48 hours, followed by extensive rinsing with high-purity deionized water. All chemicals utilized in the study were of analytical grade. Serum samples were diluted 1:10 with 0.3% HNO_3_ (Merck, Darmstadt, Germany) for analysis. Calibration standards were prepared from stock solutions at a concentration of 1,000 mg/L (Chem-Lab, Belgium). Elemental solutions with concentrations of 0.010, 0.025, 0.050, 0.100, 0.250, and 0.500 ppm were prepared using the stock solution and distilled water (Millipore, Bedford, MA, ABD) containing 0.3% HNO_3_. Selenium levels were determined by recording the absorbance at a wavelength of 196.026 nm.

### Measurement of selenoprotein levels in serum samples

Serum GPx1 and SelP levels were measured using ELISA kits (Sunred Biological Technology, Shanghai, China). All procedures were performed according to the manufacturer’s instructions.

### Measurement of cytokine levels in serum samples

The levels of IL-1β, IL-6, IL-18, and TNF-α in the serum samples were quantified using an ELISA kit specific for proinflammatory cytokines (Sunred Biological Technology, Shanghai, China) according to the manufacturer’s instructions and guidelines.

### Statistical analysis

The data are presented as the means ± SDs. All measurements were performed in triplicate and accepted when the variability between measurements was less than 5%. After the normality assumption was checked with the Shapiro-Wilk normality test, the statistical analysis was performed using one-way ANOVA and the Bonferroni post hoc correction. P < 0.05 was considered to indicate a statistically significant difference. Correlations between biochemical parameters and trace element levels were assessed by calculating Spearman’s correlation coefficients. All calculations were performed using GraphPad Prism version 5.00 for Windows (GraphPad Software, Inc., La Jolla, CA, USA).

## RESULTS

### Thyroid function markers

The serum FT3 and FT4 levels increased, but the TSH levels decreased in the HT group compared with those in the control group (P < 0.05 for all). Additionally, the FT4 levels decreased and the TSH values increased in the HT + Se 2 group compared with those in the HT group (P < 0.05 for both) (Table 1).

**Table 1:** Serum thyroid function parameters and selenium levels

	Control n=8	HT Group n=8	Se 1 Group n=8	Se 2 Group n=8	HT+Se 1 Group n=8	HT+Se 2 Group n=8
FT3 (ng/mL)	1.51±0.29	1.91±0.35^a^*	1.64±0.36	1.70±0.29	1.89±0.36	1.69±0.26
FT4 (pmol/L)	8.62±1.95	11.76±2.33^a^*	9.94±1.21	9.42±1.61	10.05±1.14	9.45±0.65^d^*
TSH (mlU/L)	0.79±0.13	0.65±0.13^a^*	0.69±0.09	0.71±0.07	0.68±0.13	0.80±0.08^d^*
Se (µg/L)	5.77±0.45	5.01±0.64^a^*	6.08±0.5	6.78±0.63	5.66±0.71^b^*	6.07±0.34^c^*

Data are means + SD; Na_2_SeO_3_: sodium selenite; HT (hyperthyroid) group (fed with 0.4 mg/100 gr L-thyroxine); Se 1 group (fed with 0.5 mg/kg Na_2_SeO_3_); Se 2 group (fed with 1 mg/kg Na_2_SeO_3_); HT + Se 1 group (0.4 mg/100 g L-thyroxine + 0.5 mg/kg Na_2_SeO_3_); HT + Se 2 group (0.4 mg/100 g L-thyroxine + 1 mg/kg Na_2_SeO_3_); aHT *vs.* Control. bSe 1 *vs.* HT + Se 1. cSe 2 *vs.* HT + Se 2. dHT *vs.* HT + Se 2. *p < 0.05.

### Serum selenium status

The serum selenium levels in the HT group were lower than those in the control group (P < 0.05). The selenium levels in the HT + Se 1 and HT + Se 2 groups were lower than those in the Se 1 and Se 2 groups (P < 0.05 for both). Selenium levels were higher in the HT + Se 2 group than in the HT group (P < 0.001) (Table 1).

### Serum selenoprotein status

Compared with the levels in the control group, GPx1 and SelP levels were significantly decreased in the HT group (P < 0.05 for both). GPx1 levels were increased in the HT + Se 2 group compared with the HT group, but their levels were decreased in the HT + Se 1 group compared to the Se 1 group and in the HT + Se 2 group compared with the Se 2 group (P < 0.05, P < 0.01, and P < 0.001, respectively). Additionally, SelP levels were decreased in the HT + Se 2 group compared with the Se 2 group (P < 0.05) (Table 2).

**Table 2:** Selenoprotein and cytokine concentrations control and experimental groups

	Control n=8	HT Group n=8	Se 1 Group n=8	Se 2 Group n=8	HT+Se 1 Group n=8	HT+Se 2 Group n=8
GPx1 (ng/mL)	23.43±2.81	17.98±3.27^a^**	23.46±3.54	26.38±3.02	18.55±1.95^d^**	21.22±1.01^c^*, ^e^***
SelP (ng/mL)	274.20±28.83	241.80±28.26^a^*	280.00±30.07	290.70±20.74	266.40±46.53	271.80±23.08^c^*
TNF-α (ng/mL)	76.73±13.22	105.60±17.76^a^**	67.94±11.14	63.49±8.96	80.69+11.08^b^***	75.25±9.81^c^**, ^e^**
IL-1β (pg/L)	978.90±188.90	1201.00±219.10^a^*	1011.00±252.00	1003.00±159.60	1086.00±166.30	1050.00±232.40
IL-6 (pg/mL)	51.39±4.48	74.99±4.44^a^*	50.36±4.75	42.38±2.10	59.71±5.12	50.64±2.80^c^**, ^e^*
IL-18 (ng/L)	21.45±3.82	27.13±3.74^a^*	21.98±4.34	21.56±2.08	25.79±4.21	23.60±2.84^c^*

Data are means ± SD; Na_2_SeO_3_: sodium selenite; HT (hyperthyroid) group (fed with 0.4 mg/100 g L-thyroxine); Se 1 group (fed with 0.5 mg/kg Na_2_SeO_3_); Se 2 group (fed with 1 mg/kg Na_2_SeO_3_); HT + Se 1 group (0.4 mg/100 gr L-thyroxine + 0.5 mg/kg Na_2_SeO_3_); HT + Se 2 group (0.4 mg/100 g L-thyroxine + 1 mg/kg Na_2_SeO_3_). ^a^HT *vs.* Control. ^b^HT *vs.* HT + Se 1. ^c^HT *vs.* HT + Se 2. ^d^Se 1 *vs.* HT + Se 1. ^e^Se 2 vs. HT + Se 2. *p < 0.05. **p < 0.01. ***p < 0.001.

### Serum cytokine status

The serum TNF-α, IL-1β, IL-6, and IL-18 levels were significantly increased in the HT group compared with the control group (P < 0.01, P < 0.05, P < 0.05, and P < 0.05, respectively). The TNF-α level decreased in the HT + Se1 compared with the HT group (p < 0.001). No significant differences in the levels of the other proinflammatory markers were observed between the HT + Se 1 and HT groups (P > 0.05 for all).

The serum TNF-α, IL-6, and IL-18 levels were significantly decreased in the HT + Se 2 group compared with the HT group (P < 0.01, P < 0.01, and P < 0.05, respectively). Although a statistically significant difference in the IL-1β levels was not observed, its levels were reduced in the HT + Se 2 group compared with the HT group. Additionally, TNF-α and IL-6 levels were significantly increased in the HT + Se 2 group compared with the Se 2 group (P < 0.01 and P < 0.05, respectively). No significant differences in the other parameters were observed between the HT + Se 2 group and the Se 2 group (Table 2).

### Correlation analysis

Positive correlations were identified between FT3 and FT4 levels, between FT3 and IL-6 levels, between FT3 and IL-18 levels, between FT4 and TSH levels, between FT4 and IL-6 levels, between TSH and IL-6 levels, and between TSH and TNF-α levels in the HT group (r = 0.715, r = 0.756, r = 0.710, r = 0.731, r = 0.943, r = 0.760, and r = 0.722, respectively).

The results of the correlation analysis of the HT + Se 1 group revealed positive correlations between FT4 and TSH levels, FT4 and IL-1β levels, SelP and IL-1β levels, Se and IL-1β levels, and Se and IL-6 levels (r = 0.799, r = 0.740, r = 0.716, r = 0.747, and r = 0.749, respectively), but a negative correlation was observed between GPx1 and TNF-α levels (r = −0.729).

Additionally, the results of the correlation analysis of the HT + Se 2 group indicated positive correlations between TSH and IL-6 levels, SelP and IL-1β levels, SelP and IL-6 levels, and SelP and TNF-α levels (r = 0.777, r = 0.893, r = 0.745, and r = 0.793, respectively), but a negative correlation was observed between Se and GPx1 levels (r = −0.931) (Table 3).

**Table 3:** Correlation results of HT, HT + Se 1, and HT + Se groups

Parameters	HT group	Parameters	HT+Se 1 Group	Parameters	HT+Se 2 Group
FT3 *vs.* FT4	P = 0.046	FT4 *vs.* TSH	P = 0.017	TSH *vs.* IL-6	P = 0.023
	r = 0.715		r = 0.799		r = 0.777
FT3 *vs.* IL-6	P = 0.029	FT4 vs. IL-1 β	P = 0.035	SelP *vs.* IL-1β	P = 0.003
	r = 0.756		r = 0.740		r = 0.893
FT3 *vs.* IL-18	P = 0.049	SelP vs. IL-1 β	P = 0.045	SelP *vs.* IL-6	P = 0.033
	r = 0.710		r = 0.716		r = 0.745
FT4 *vs.* TSH	P = 0.039	GPx1 *vs.* TNF-α	P = 0.039	SelP *vs.* TNF-α	P = 0.018
	r = 0.731		r = −0.729		r = 0.793
FT4 *vs.* IL-6	P < 0.001	Se *vs.* IL-1β	P = 0.033	Se *vs.* GPx1	P = 0.001
	r = 0.943		r = 0.747		r = −0.931
TSH *vs.* IL-6	P = 0.028	Se *vs.* IL-6	P = 0.032		
	r = 0.760		r = 0.749		
TSH vs. TNF-α	P = 0.042				
	r = 0.722				

HT (hyperthyroid) group (fed with 0.4 mg/100 g L-thyroxine); HT + Se 1 group (0.4 mg/100 g L-thyroxine + 0.5 mg/kg Na_2_SeO_3_); HT + Se 2 group (0.4 mg/100 g L-thyroxine+1 mg/kg Na_2_SeO_3_); r, Correlation coefficient; P, Significance level.

## DISCUSSION

Hyperthyroidism is a common condition with a prevalence of 0.2%-1.3% worldwide and can lead to serious health consequences. This disease is associated with an increased metabolic rate, increased oxygen consumption, dysfunction of the mitochondrial respiratory chain, increased intracellular ATP consumption, and increased production of reactive oxygen species (ROS) (^15,24^). The increased ROS production in individuals with hyperthyroidism results in oxidative stress. Recent findings suggest that mitochondrial reactive species act as signalling molecules that mediate the production of proinflammatory cytokines, thereby linking oxidative stress and inflammation. However, the effects of hyperthyroidism on antioxidant enzyme activity depend on the specific tissue examined (^25^).

Selenium, which plays roles in proper thyroid metabolism and intracellular redox control, is a crucial component of selenoprotein synthesis (^15^). Additionally, selenium deficiency has been reported to affect pathological thyroid morphology, and selenium supplementation has been shown to have some protective effects on pathological disturbances (^26^). Selenium supplementation in individuals with hyperthyroidism provides antioxidant and anti-inflammatory benefits by reducing oxidative stress through its role as a cofactor for enzymes such as glutathione peroxidase and thioredoxin reductase (^27^). In individuals with autoimmune hyperthyroidism, particularly Graves’ disease, it lowers thyroid autoantibody levels (TRAb and TPOAb) and supports thyroid hormone regulation by facilitating the T4-to-T3 conversion and controlling free radicals (^28,29^). Additionally, selenium ameliorates Graves’ orbitopathy by alleviating eye symptoms, improving quality of life (^30^).

Selenium is essential in trace amounts for human and animal health, but it becomes harmful in excess quantities. The narrow range between selenium deficiency (<40 µg/day) and toxicity (>400 µg/day) necessitates careful control of its intake (^26^). In the context of selenium supplementation for hyperthyroidism, caution must be exercised to avoid excess selenium intake, as prolonged exposure to high doses can lead to toxicity. Short-term use (3-6 months) at 100-200 µg/day is effective for its antioxidant benefits and ability to reduce antibody levels, whereas long-term use (up to 12 months) may be required for chronic complications, necessitating regular monitoring to prevent toxicity (^30,31^). Exceeding the upper tolerable intake level for selenium over time may increase the risk of toxicity (^32^). Therefore, selenium levels must be monitored and the dosages must be adjusted accordingly to ensure that supplementation is effective without exceeding safe limits. Considering this information, our study assessed the effects of selenium supplementation at two different doses on the levels of thyroid hormones, proinflammatory cytokines, and selenoproteins, aiming to determine the optimal selenium dose for supplementation in a hyperthyroid rat model.

Hyperthyroidism can be experimentally induced in animal models through the administration of exogenous levothyroxine (T4) or thyroid-stimulating agents, which replicate the metabolic effects but lack the immunological components of autoimmune hyperthyroidism (^33^). Selenium supplementation helps regulate metabolism in artificial hyperthyroidism models and exerts immunomodulatory effects on autoimmune cases, balancing immune responses by reducing the levels of proinflammatory cytokines (^29,34^). Additionally, selenium protects thyroid function by mitigating follicular damage and apoptosis (^35^). Thus, its benefits are likely more significant in autoimmune hyperthyroidism models than in experimentally induced models.

A review of studies on selenium levels in individuals with hyperthyroidism revealed that the majority of these studies were conducted in humans. Arikan reported that maternal serum selenium levels were lower in pregnant women with hyperthyroidism than in healthy pregnant women. These findings support the hypothesis that reduced selenium levels in individuals with hyperthyroidism are related to an impaired antioxidant response (^36^). Similarly, Bülow Pedersen and cols. observed lower selenium levels in Graves’ disease patients than in control individuals in their study of the Danish population (^37^). Wertenbruch and cols. compared mean serum selenium levels between the remission and relapse groups of patients with Graves’ disease and detected the highest serum selenium levels (>120 µg/L) in the remission group, suggesting that selenium levels have a positive effect on the outcome of Graves’ disease (^38^). Our findings, which are consistent with the literature, indicate that serum selenium levels are reduced in individuals with hyperthyroidism (p < 0.05). These data suggest that selenium supplementation may be beneficial in the treatment of hyperthyroidism.

Numerous studies have examined the effectiveness of selenium supplementation in the treatment of hyperthyroidism, but the results of these studies have not always been consistent. Zheng and cols. reported that in Graves’ disease patients receiving selenium supplementation, the levels of FT4 and FT3 were significantly lower than those in the placebo group at 3 months, with these findings being consistent at 6 months but not at 9 months. Additionally, the selenium group presented higher TSH levels than the control group at 3 and 6 months, although this difference was not observed at 9 months (^16^). Wang and cols. evaluated the effectiveness of selenium supplementation in patients with recurrent Graves’ disease and reported that FT4 and FT3 levels decreased at 2 months in a group receiving methimazole and selenium compared with a group receiving only methimazole, whereas TSH levels increased (^39^). Another study showed that a treatment combining methimazole with antioxidants containing selenium (83 µg of selenomethionine + 17 µg of selenium yeast + vitamin D) was more effective at improving thyroid activity than methimazole alone (^40^). In a study by Nordio, patients taking 500 mg of L-carnitine and 83 µg of selenium in a tablet daily for 1 month presented a significant reduction in symptoms related to subclinical hyperthyroidism and improved quality of life, although no significant impact on thyroid function was observed (^17^). Leo and cols. reported that selenium supplementation did not provide any advantage in the short-term management of hyperthyroidism when selenium intake was adequate. They emphasized the need to evaluate an individual’s selenium status before starting antithyroid medication to determine if selenium supplementation would be beneficial (^18^). Our findings indicate that in the HT group, the serum FT3 and FT4 levels were higher than those in the control group, whereas the TSH levels were lower. Compared with those in the HT group without selenium supplementation, the FT3 and FT4 levels decreased in the HT group with 1 mg/kg sodium selenite added to the diet, whereas the TSH levels increased. These results suggest that selenium supplementation effectively regulates thyroid hormones.

Cytokines are proteins secreted by immune and endocrine cells that regulate various immunological, inflammatory, and endocrine processes (^41^). These molecules play a role in the pathogenesis of thyroid diseases by functioning within the immune system and directly targeting thyroid follicular cells. They participate in both the induction and effector phases of the immune response and inflammation, playing a key role in the pathogenesis of autoimmune thyroid diseases, including Graves’ disease. The presence of multiple cytokines (IL-1α, IL-1β, IL-2, IL-4, IL-6, IL-8, IL-10, IL-12, IL-13, IL-14, TNF-α, and IFN-γ) in inflammatory cells and thyroid follicular cells has been documented (^42^).

Reviewing studies on the relationship between hyperthyroidism and cytokines in the literature, Akber and cols. reported increased levels of IL-6, IL-18, and TNF-α in hyperthyroid patients, suggesting that proinflammatory cytokines may play a role in the pathogenesis of this condition (^19^). Jha and cols. observed elevated circulating IL-6 levels in women with hyperthyroidism, irrespective of the aetiology of thyroid hyperfunction. They reported that increased serum IL-6 levels in patients with hyperthyroidism could originate from various sources, including the thyroid gland, mononuclear blood cells, and bone tissue (^43^). Another study revealed that serum IL-6 concentrations were significantly higher in both untreated and methimazole-treated patients with Graves’ disease than in controls, with serum sIL-6R concentrations being significantly affected by treatment, whereas IL-1Ra concentrations did not differ between treated or untreated patients with Graves’ disease and controls (^44^). Ferrari and cols. reported that cytokine levels are elevated in patients with autoimmune and non-autoimmune hyperthyroidism, suggesting that this increase may be due more to the chronic effects of excess thyroid hormones than to the inflammatory state itself (^45^).

Our findings revealed that in the HT group, the serum levels of the cytokines TNF-α, IL-1β, IL-6, and IL-18 were elevated compared with those in the control group. Compared with those in the HT group, the cytokine levels in the HT group fed a diet containing 0.5 mg/kg sodium selenite (HT + Se 1 group) were lower, although the changes were not significant except for the TNF-α level. Additionally, compared with the levels in the HT group, the levels of TNF-α, IL-6, and IL-18 in the HT group fed a diet containing 1 mg/kg sodium selenite (HT + Se 2 group) were significantly lower (Table 2). These findings indicate that cytokine levels are increased in individuals with hyperthyroidism and that selenium supplementation reduces the levels of these cytokines. These results support the hypothesis that selenium supplementation has regulatory effects on inflammation. Additionally, the positive correlations between SelP and IL-1β, IL-6, and TNF-α levels may suggest that elevated SelP levels in the HT + Se 2 group are part of the body’s response to an increased inflammatory state, highlighting its role in managing oxidative stress and inflammation (Table 3).

This study also examined the levels of selenoproteins in rats with hyperthyroidism and following selenium supplementation. Selenoproteins play crucial roles in regulating thyroid hormone metabolism and the antioxidant defence system. Selenium performs its functions after being incorporated into various selenoproteins (^18^). One such selenoprotein, GPx1, protects against oxidative stress by reducing H_2_O_2_ and lipid hydroperoxides. A reduction in seleniumdependent GPx activity can lead to the spread of H_2_O_2_ to the thyroid parenchyma, which is followed by local inflammation and eventual tissue damage (^4^). SelP plays a significant role in thyroid hormone metabolism by assisting in the synthesis and processing of the deiodinases required to convert T4 into T3. Selenium supplementation has been shown to significantly increase the activity of GPx, an important antioxidant enzyme, leading to improved outcomes of various health conditions characterized by oxidative stress. Studies indicate that selenium is a crucial cofactor for GPx enzymes and that optimal selenium levels can increase their activity, leading to more effective antioxidant defences (^46^). Research has shown that individuals receiving adequate selenium supplementation have better GPx enzyme activity, which is associated with reduced oxidative damage and improved cellular function (32). Therefore, selenium supplementation that supports maximum GPx activity is essential for optimizing its health benefits.

Studies examining the relationship between hyperthyroidism and selenoprotein levels have produced conflicting results. Federige and cols. reported lower serum SelP levels in patients with hyperthyroidism than in controls. These findings suggest that the reduced serum SelP levels in hyperthyroid patients indicate increased consumption of selenium-dependent proteins to counteract inflammatory reactions and free radicals resulting from autoimmune attacks (^20^). Mayer and cols. measured GPx activity in individuals with hyperthyroidism and found that it was lower than the level in the control group (^47^). However,

Andryskowski and cols. reported that erythrocyte GPx activity was higher in hyperthyroid patients than in controls (^21^). Bednarek and cols. detected lower GPx activity in untreated patients with Graves’ disease than in controls, but GPx activity increased after approximately 3 months of treatment, with no significant change compared with controls (^48^). In contrast, Rybus-Kalinowska and cols. evaluated erythrocyte GPx levels in hyperthyroid patients before and after treatment and reported a decrease following treatment (^49^). Our study revealed that the serum levels of GPx1 and SelP were lower in the HT group than in the control group. In the HT group fed a diet containing 0.5 mg/kg sodium selenite (HT + Se 1 group), selenoprotein levels increased compared with those in the HT group, although this change was not significant. Compared with those in the HT group, significantly increased GPx1 and SelP levels were detected in the HT group fed a diet containing 1 mg/kg sodium selenite (HT + Se 2 group). Our findings suggest that selenoprotein levels are reduced in individuals with hyperthyroidism and that selenium supplementation increases these levels. These results support the hypothesis that selenium exerts regulatory effects on oxidative stress. Given that selenium is a critical component of the antioxidant enzyme GPx1, the observed negative correlation between selenium and GPx1 levels in the HT + Se 2 group may indicate that selenium is utilized to modulate GPx1 levels (Table 3).

Selenium supplementation may be influenced by genetic polymorphisms in *GPX* genes, which play crucial roles in antioxidant defences. Research suggests that variations in the *GPX1, GPX2,* and *GPX4* genes can affect individual responses to selenium supplementation. For example, the Pro198Leu variant in the *GPX1* gene is associated with changes in selenium utilization and antioxidant enzyme activity (^2^,^50^). Individuals with specific polymorphisms may require different selenium doses to achieve optimal antioxidant effects, as these genetic variations can influence GPx enzyme expression and activity (^32^).

In conclusion, our findings indicate that selenium and selenoprotein levels are reduced in a rat model of hyperthyroidism, whereas the levels of proinflammatory markers are increased. Specifically, oral supplementation with 1 mg/kg sodium selenite appears to be useful in regulating the disrupted balance of selenoproteins and inflammation in an experimental hyperthyroidism model.
